# Supplementary figures and images for: Combined reference-free and multi-reference based GWAS uncover cryptic variation underlying rapid adaptation in a fungal plant pathogen
Source: PLoS Pathog. 2023 Nov 16;19(11):e1011801. doi: 10.1371/journal.ppat.1011801 (PMC10688896; doi:10.1371/journal.ppat.1011801)

**A**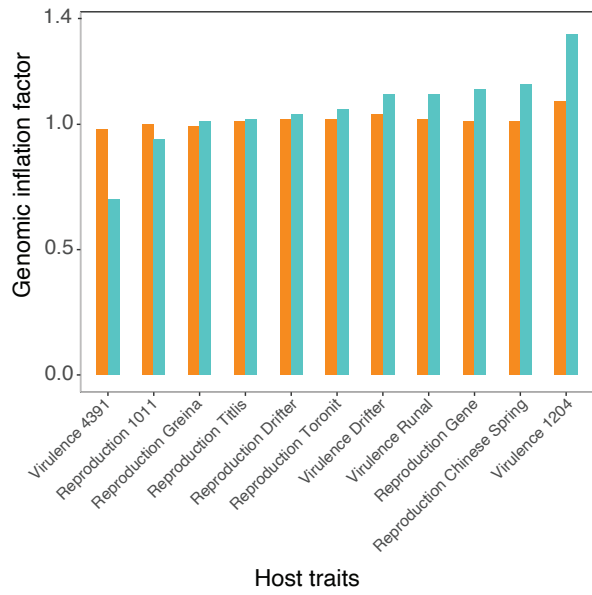**B**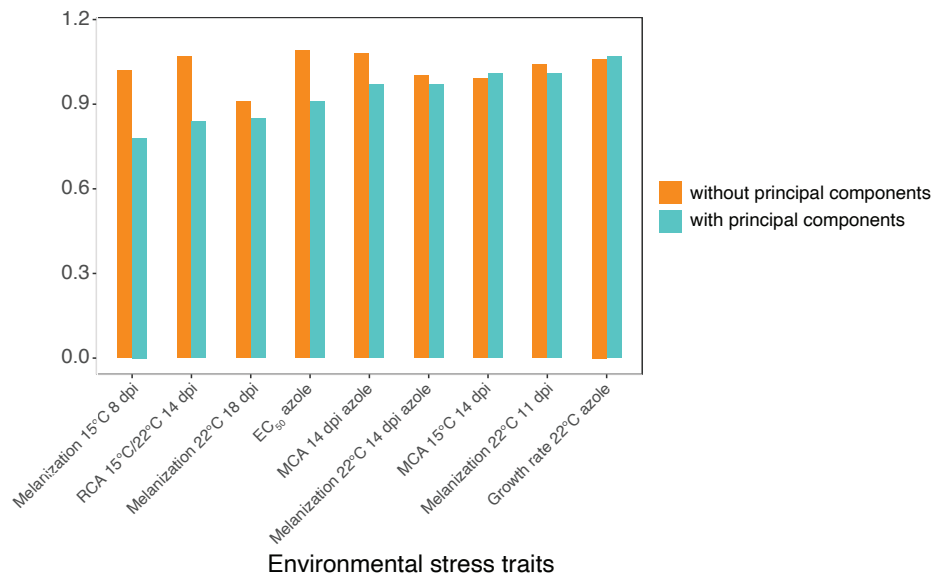

Supplement: S1 Fig — (A) host-related traits i.e. pathogen virulence (percentage of the leaf surface covered by necrotic lesions) and reproduction (pycnidia density within lesions) and (B) environmental stress related traits. Pathogen virulence and reproduction were measured on 12 genetically diverse wheat lines. (PDF) [file ppat.1011801.s001.pdf]

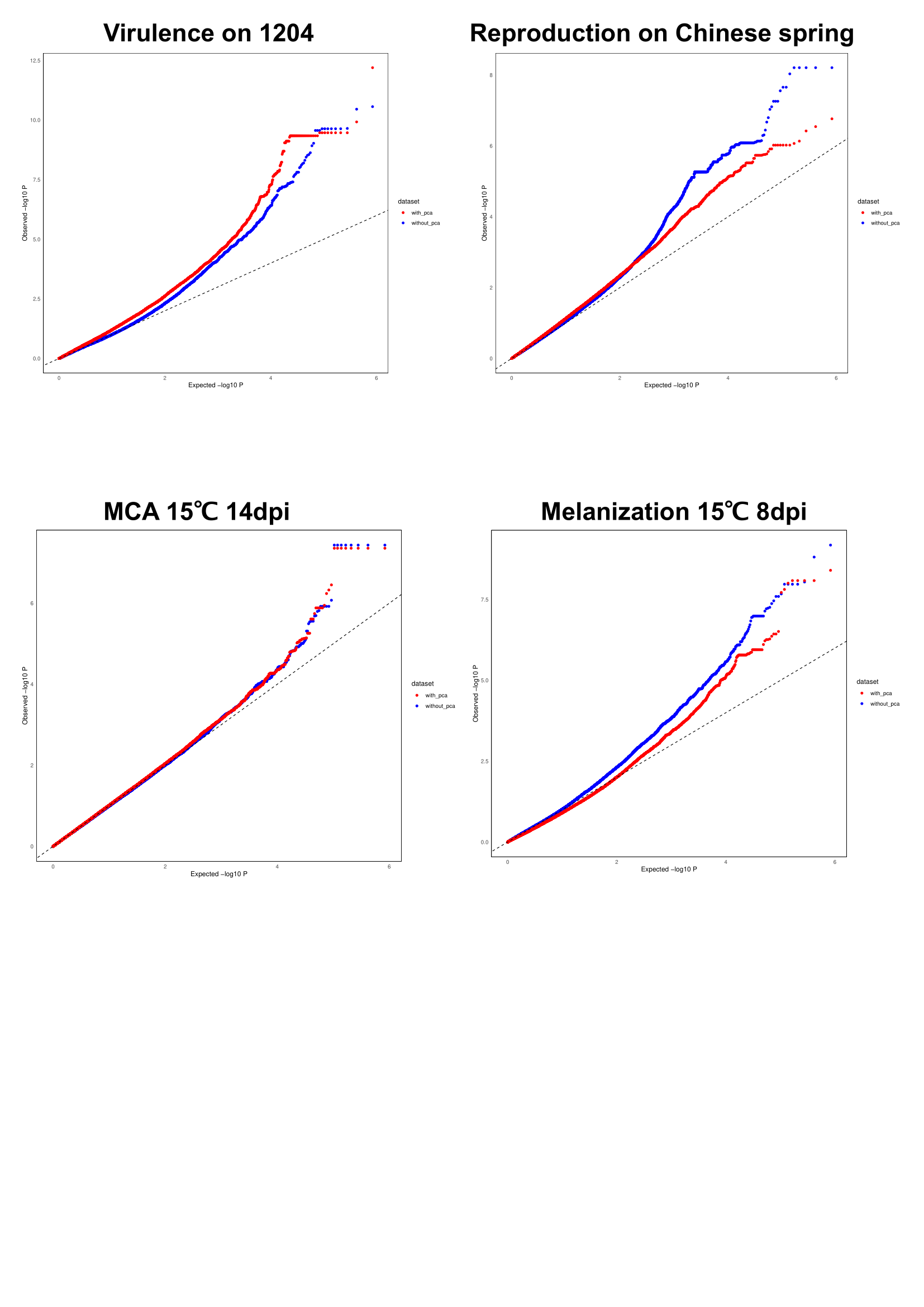

Supplement: S2 Fig — Red dots indicate P-values estimated with the first three principal components as covariates in the GWAS and blue dots indicate P-values estimated without principal components. (TIFF) [file ppat.1011801.s002.tiff]

**A**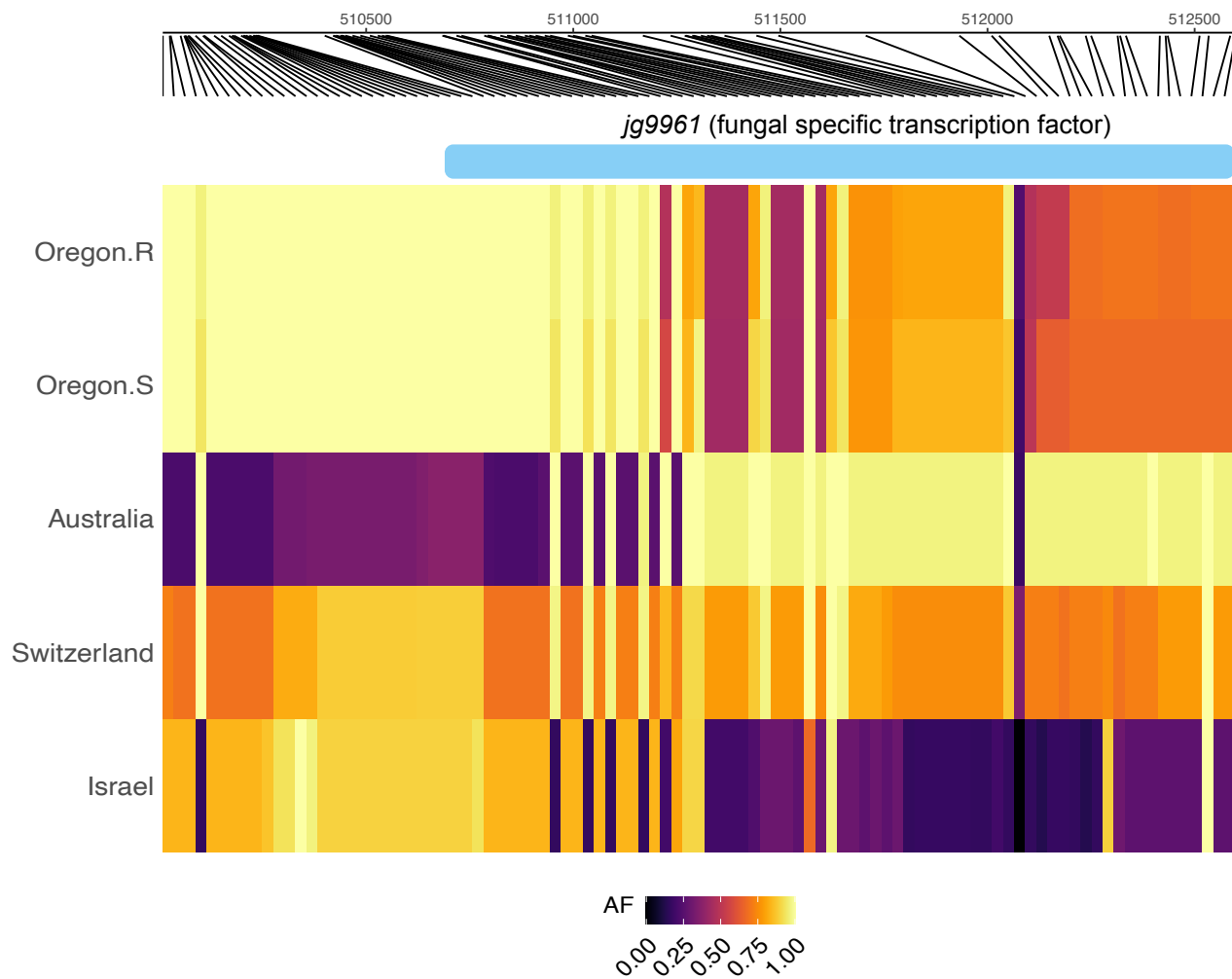**B**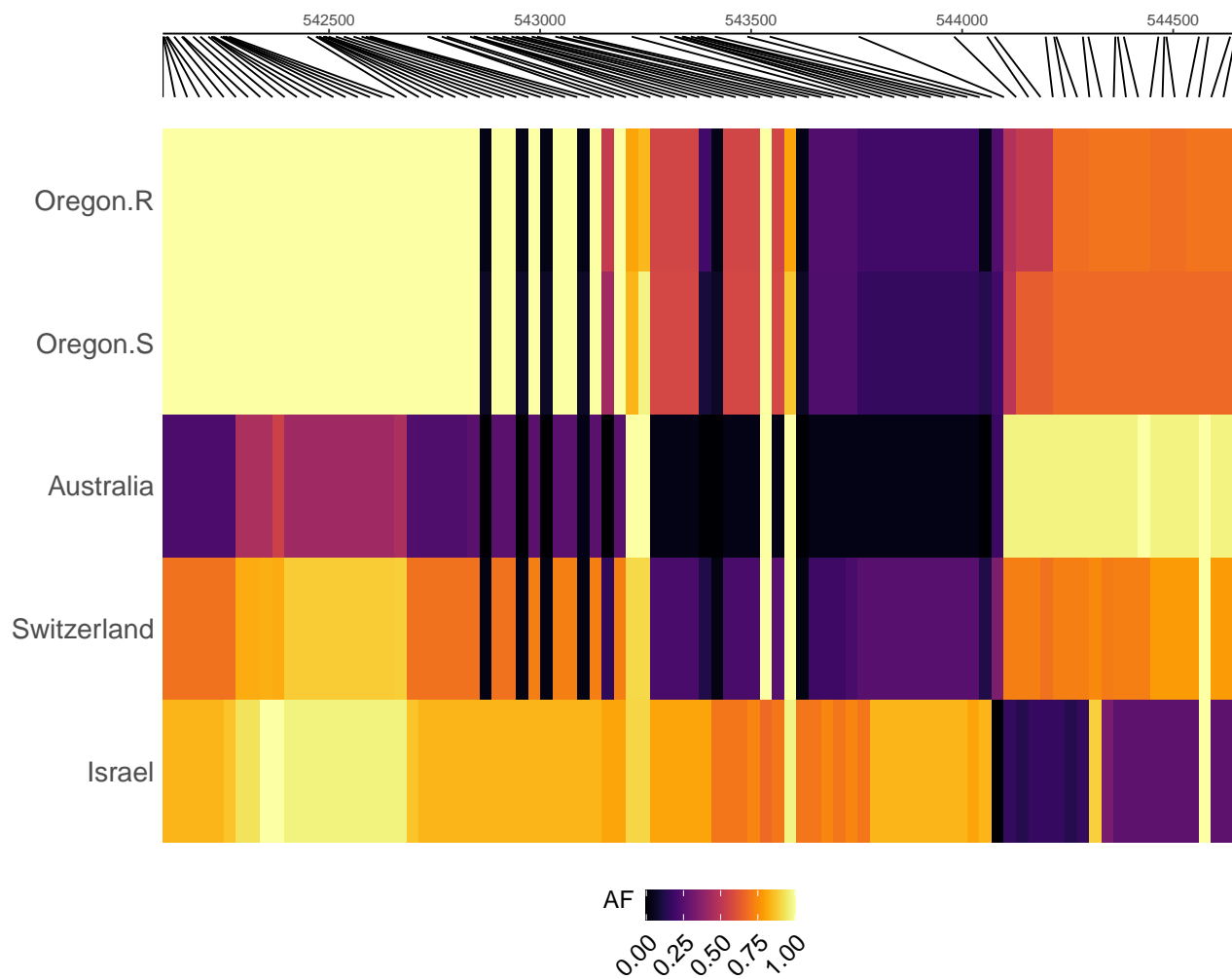

Supplement: S3 Fig — For (A) reference genome KE94 and (B) reference genome IPO323. The color gradient indicates the estimated allele frequency of each SNP in the respective position. A frequency of 0 indicates the allele is absent and 1 indicates the allele is fixed. (PDF) [file ppat.1011801.s003.pdf]

A

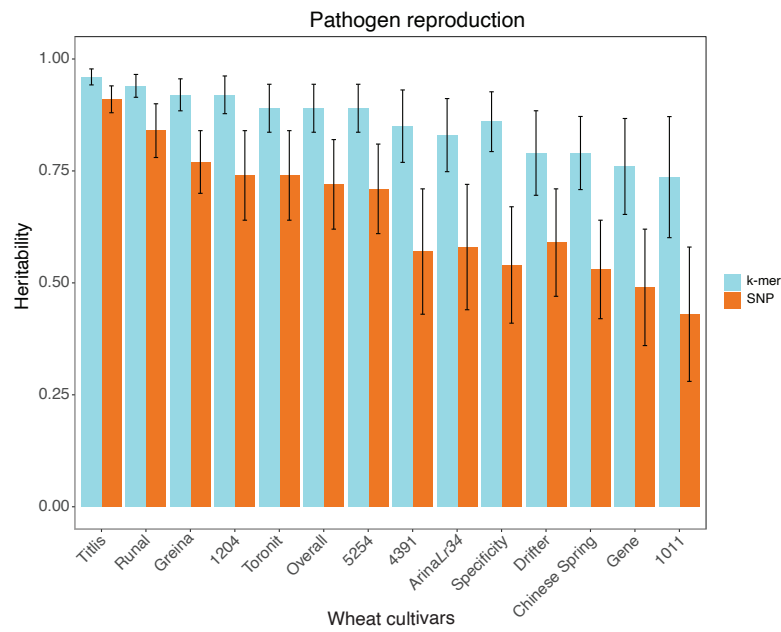

B

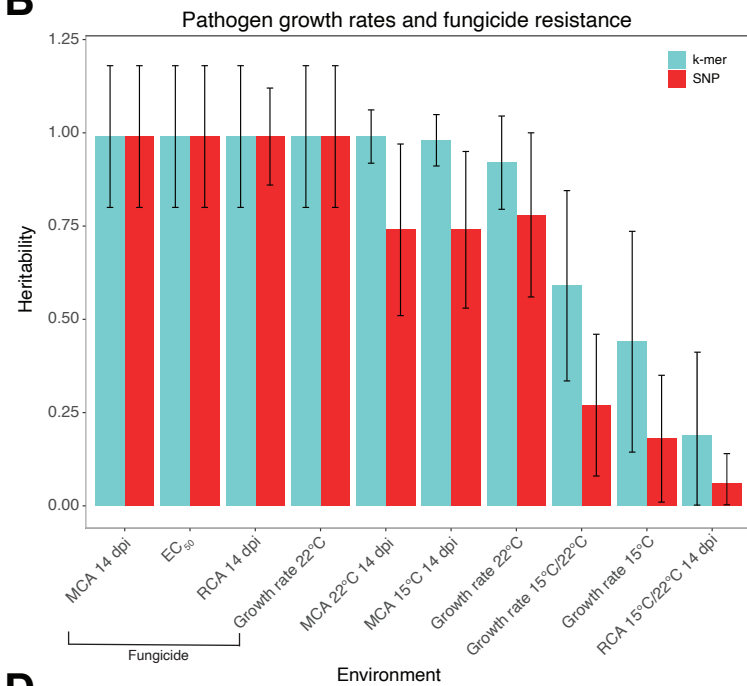

C

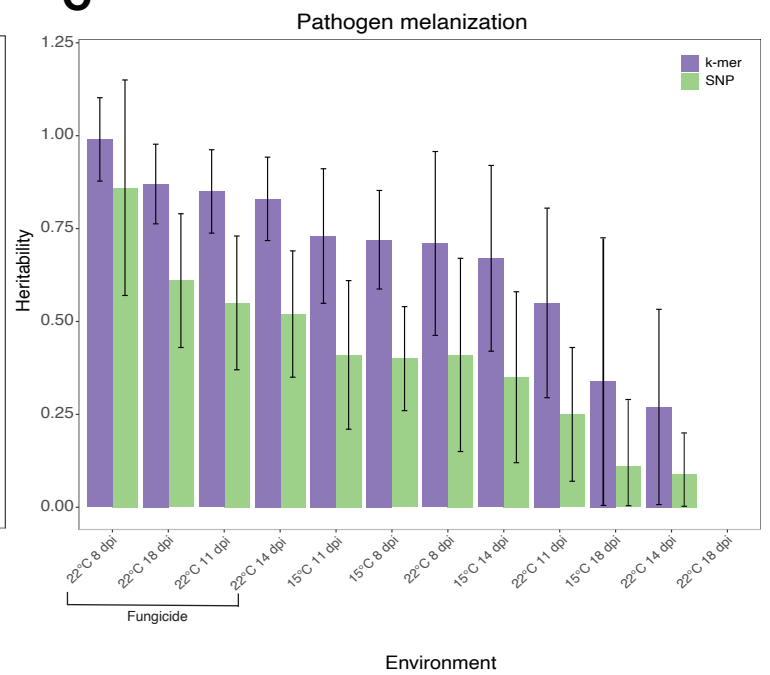

D

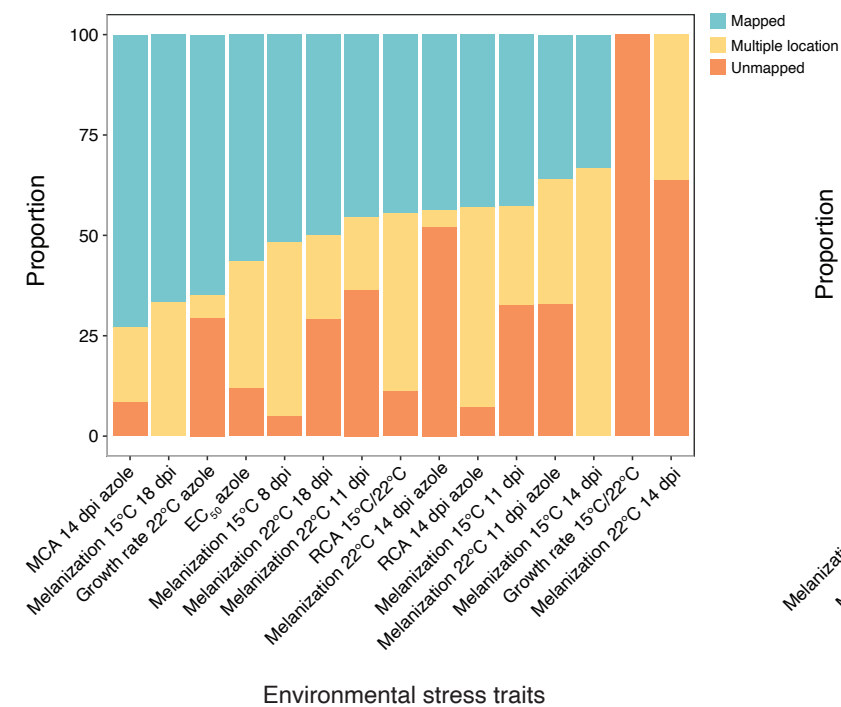

E

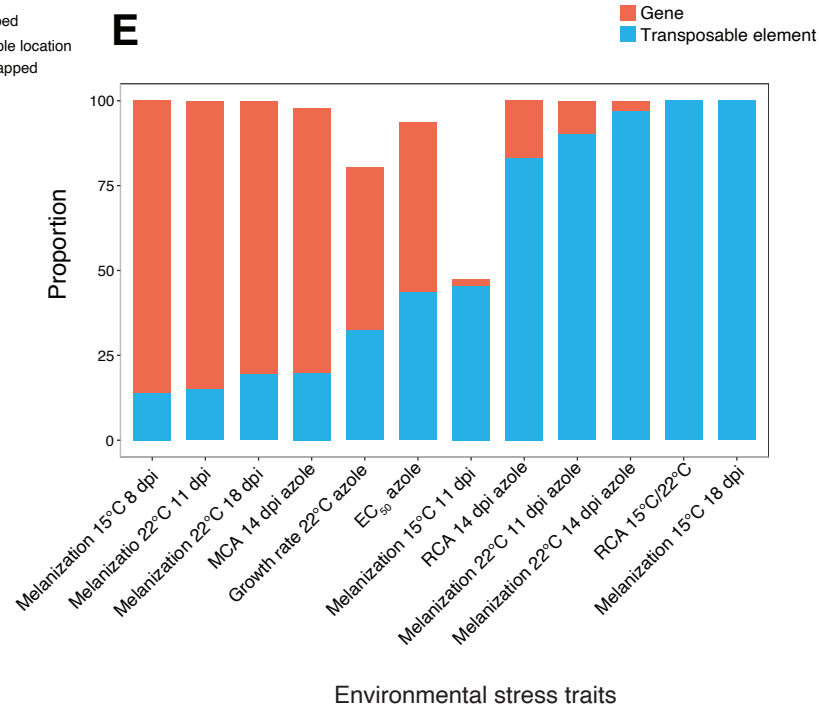

MCA=Mean colony area  
RCA=Ratio of colony area

Supplement: S4 Fig — (A) Pathogen reproduction (pycnidia density within lesions), (B) pathogen growth rate and fungicide resistance, (C) pathogen melanization. Pathogen reproduction was measured on 12 genetically diverse wheat lines. Overall reproduction represents the average value of reproduction measured on 12 genetically diverse wheat lines. Reproduction specificity was estimated based on the adjusted coefficient of variation of mean reproduction across 12 genetically diverse wheat lines. Higher specificity suggests affinity to certain hosts for maximizing reproductive fitness. Both SNP-based and K-mer-based heritability were estimated by following a genome-based restricted maximum likelihood (GREML) approach. Standard errors are indicated by error bars. (D) Alignment of significantly associated K-mers against the reference genome (IPO323) show the proportion of K-mers having a unique mapping position, multiple locations, or no unambiguous mapping position in environmental stress-related traits. (E) Proportion of significant K-mers with a unique mapping position in the reference genome either tagging a gene or a transposable element in environmental stress-related traits. (PDF) [file ppat.1011801.s004.pdf]

**A**

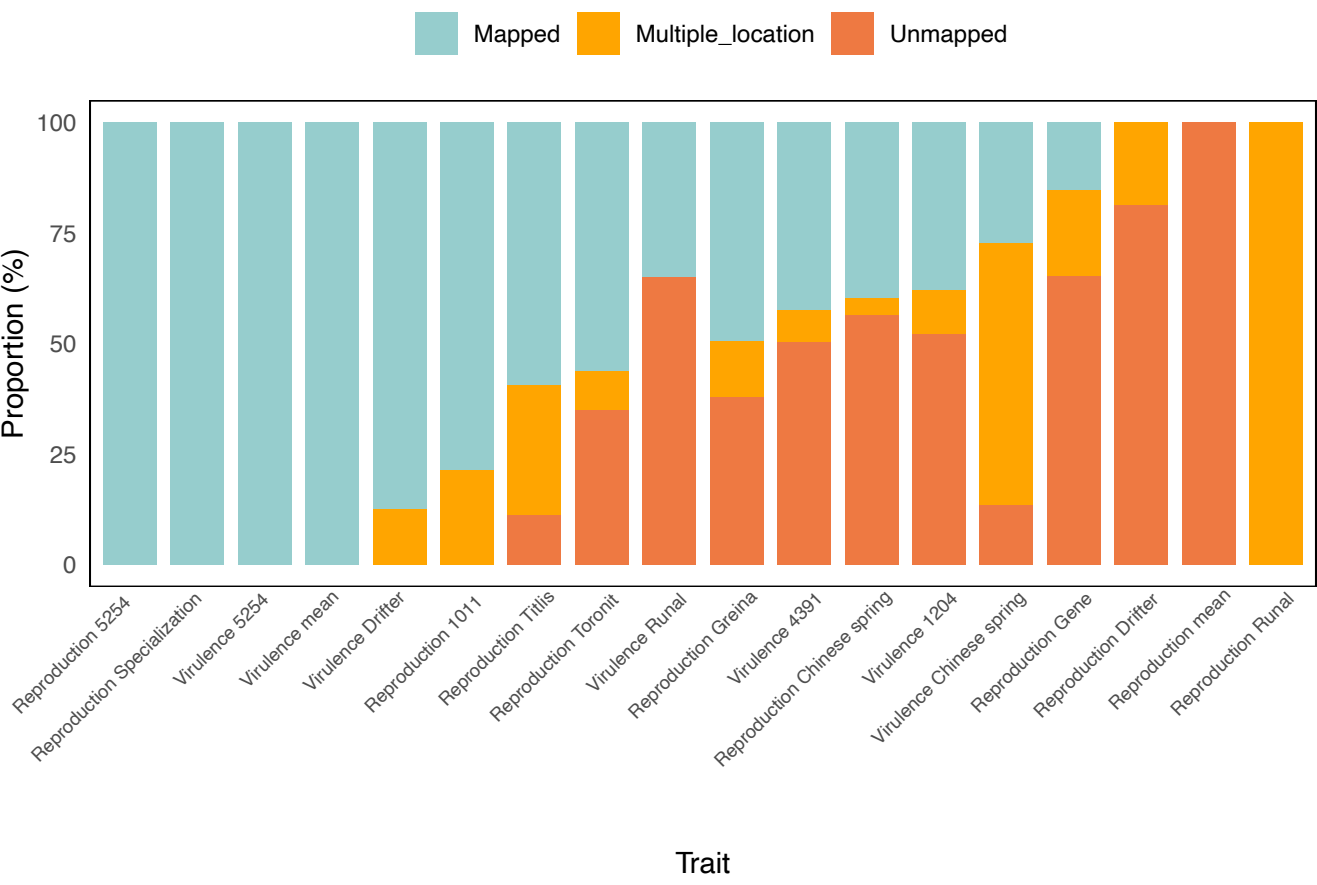

**B**

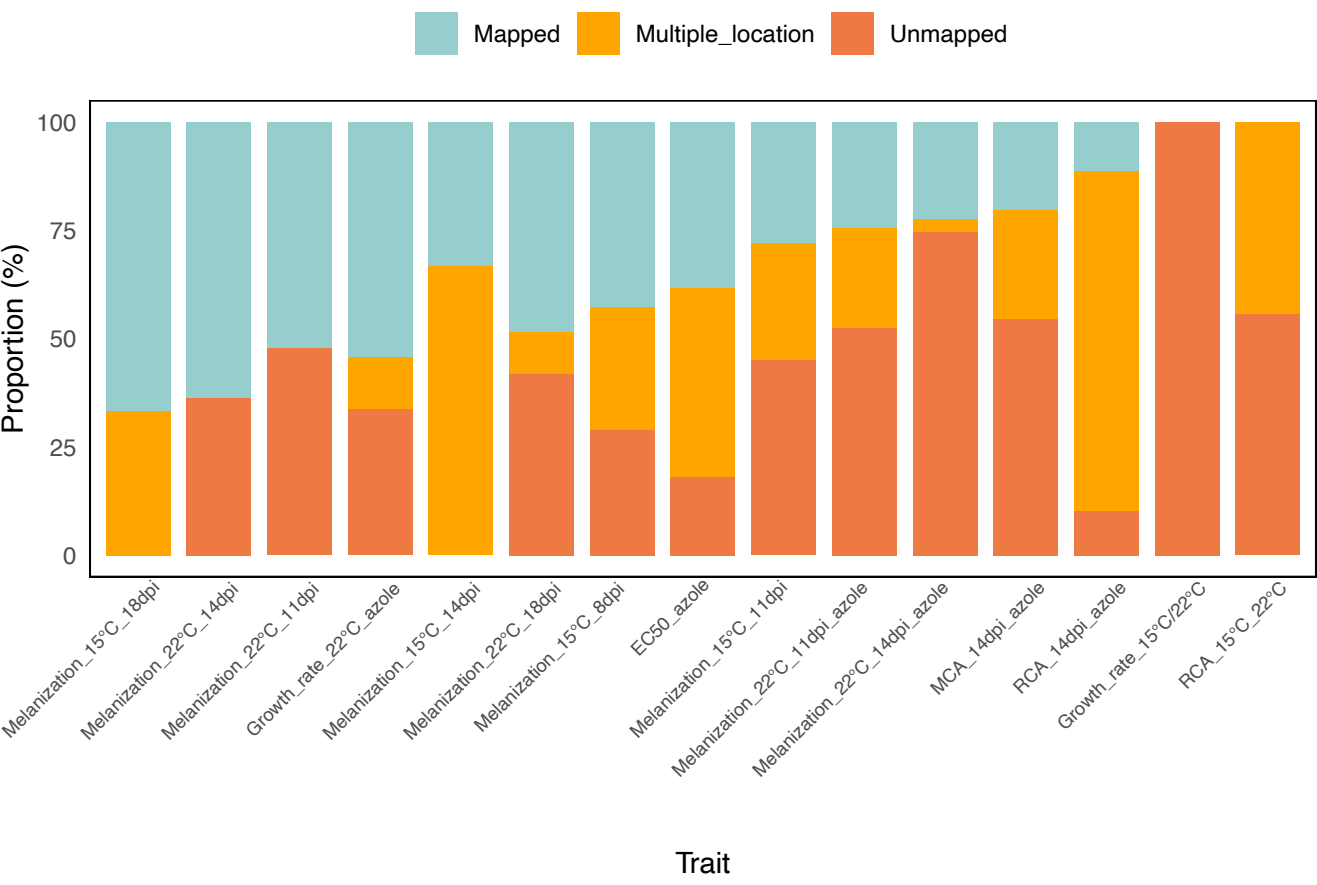

Supplement: S5 Fig — Proportion of k-mers having a unique mapping position, multiple locations, or no unambiguous mapping position in (A) Host related and (B) environmental stress-related traits. (PDF) [file ppat.1011801.s005.pdf]

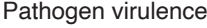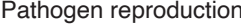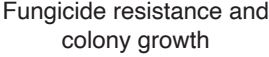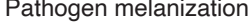

Supplement: S6 Fig — The intersection size is the number of genes and the black dots on the matrix represent whether the genes are shared or unique to different reference genomes and the K-mer approach. For example, the first vertical bar in each graph shows the number of genes that are uniquely identified by the K-mer GWAS, while the last vertical bar demonstrates the number of genes that are commonly identified by all the reference-based and K-mer GWAS. Pathogen virulence and reproduction were measured on 12 genetically diverse wheat lines. (PDF) [file ppat.1011801.s006.pdf]
