# Supplementary material for: Combined reference-free and multi-reference based GWAS uncover cryptic variation underlying rapid adaptation in a fungal plant pathogen
Source: PLoS Pathog. 2023 Nov 16;19(11):e1011801. doi: 10.1371/journal.ppat.1011801 (PMC10688896; doi:10.1371/journal.ppat.1011801)
Supplement: Attachment — Submitted filename: Response_reviewers.docx [file ppat.1011801.s008.docx]

*Dear Dr. Croll,

The submitted manuscript has undergone peer review by three independent experts. The reviewer’s all commented on the value of the analysis, the demonstration of bias in single-reference GWAS studies, and that while the approach is not entirely new, it is a good demonstration and guide-post for use in fungal systems.

There are questions raised by the authors that require addressing and warrant the decision of major revision. Some reviewers suggested the revisions could be considered minor, which can be interpreted that the authors did not think major new analyses are necessary, but that more explanation, justification, and discussion are warranted. Please pay attention to the comment by reviewer 2 asking for specific examples demonstrating PAV as causative for trait association differences, and comments by all authors regarding statistical approaches and justification.*

**RESPONSE:**

Dear Editor,

We are very grateful for the thorough and very constructive reviews. We addressed all raised issues below and detail our changes. We are convinced that this has significantly improved the message and clarified outstanding questions.

With best regards,

Daniel Croll

*Reviewer's Responses to Questions*

***Part I - Summary*** *Please use this section to discuss strengths/weaknesses of study, novelty/significance, general execution and scholarship.*

*Reviewer #1: The manuscript entitled “Combined reference-free and multi-reference based GWAS uncover cryptic variation underlying rapid adaptation in a fungal plant pathogen” collected 49 phenotypic traits of 145 strains of the fungal wheat pathogen Zymoseptoria triticim, and genotyped these strains through whole genome sequencing. Genome-wide association studies (GWAS) were conducted using a traditional SNP-based approach along with multiple reference genomes and a k-mer method to analyze various collected traits. The results revealed significantly associated SNPs, k-mers, and candidate genes. While GWAS has been extensively utilized in genetic research involving humans, animals, and plants, its application in microorganisms is relatively limited. Therefore, the utilization of GWAS, particularly k-mer GWAS given the prevalence of structural variation among Z. triticim fungal genomes, holds significant value. This study devoted significant efforts to data collection, and the methods employed for data analysis are well-described, providing ample detail. A few comments are provided for the authors' consideration.*

*Reviewer #2: Dutta et al. report the application of innovative association mapping techniques to discover hidden variation in Zymoseptoria tritici associated with virulence/ecological traits. As fungal pathogens often have highly variable genomes within a species including structural variation, classic GWAS methods that rely on SNPs called from a single reference genome inherently miss much of the standing variation, which can fail to detect true genetic associations. This manuscript takes a comprehensive approach using a vast phenotypic dataset to assess the use of SNPs called from multiple reference genomes, as well as a reference-free k-mer approach. Overall, the manuscript is very well written. The methods used are clearly described and the conclusions are generally well-founded. These results advance our understanding of functional genomic variation in fungi and provide a framework that can be applied in other systems. I have identified a couple of areas that could be expanded and some relatively minor points that I feel could bolster some conclusions and increase the clarity of the results.*

*Reviewer #3: The authors use a variety of their previously published population-level data from Z. tritici (isolate WGS, several reference genome assemblies, phenotype data) in order to address an important issue - for species with abundant structural variation and differences in gene and marker presence-absence, how much is missed in association mapping studies when defining markers solely based on a single reference genome? They compare the performance of GWAS using SNPs called with respect to the single canonical reference genome to GWAS with SNPs called with respect to an additional 18 reference genomes as well as to GWAS using a reference-genome free k-mer approach. Not surprisingly, they find that the reliance on just a single canonical reference genome generally leads to missing the identification of other significant trait-associated markers that are absent or cannot be called with the canonical reference. The k-mer GWAS approach has already been demonstrated in plant and bacterial species, this is a novel and significant demonstration of the utility of this approach in fungi, in particular in an important plant pathogenic fungus.

The manuscript is generally well-written and executed, though some areas require some clarification or revision (see below). A strength of the study is the availability of so much data from their previous studies, such that this study can focus on a comparison of analysis methods and remain fairly concise. The authors do a great job of providing important data in supplemental tables so the reader can follow along with their conclusions well. The most significant weaknesses - described immediately below - are probably something the authors can address simply - either by providing a little more information and interpretation or a bit of justification of some choices they have made in their analyses.*

*The manuscript refers in the abstract and other places to a set of 49 traits for GWAS, but then results of GWAS are only shown for 20 or so different traits. It is not clear why this is so, and should be explained - the biggest concern without any additional explanation is that the authors chosen subset of traits are not representative of the full results in some important aspects that could affect the study's conclusions. (more related comments on this issue in PartII of review).*

**RESPONSE:** We agree that the presentation of the GWAS on different traits was not well enough explained. We have provided now a clearer explanation for some ambiguous traits, mentioned that only 20 traits for which significant GWAS associations were detected and a documentation of all the 49 traits as a Supplementary Table S5. We believe that focussing on presenting the ~40% of the phenotypic traits showing some significant associations out of the total available dataset is more transparent than only mentioning the significant traits. The fact that not all traits revealed significant associations could be due to a multitude of reasons including a highly polygenic architecture of the trait.

*I agree with the authors on the relative merits of the k-mer approach in contrast to performing GWAS separately against multiple SNP sets each called against a different reference. The latter approach clearly entails a lot of redundancy (most of the SNPs are presumably redundant across most of the SNP sets).*

**RESPONSE:** We agree that multi-reference genome SNP calling entails redundancies and, hence, issues with multiple testing. We are grateful that this point is brought up. To add to this complication, the k-mer approach also entails issues of redundancy as multiple k-mer presence/absence variants can stem from the same polymorphism (*i.e.* multiple k-mers overlapping the same polymorphic locus). Hence, identifying the exact level of redundancy in either multi-reference mapping and k-mer analyses is very challenging given the difficulty in identifying orthologous positions at bp-resolution. We now clearly state limitations and propose potential remedies in the revised Discussion.

*The other major weakness in the study has to do with the tough statistical question related to the approach of using multiple SNP sets. How to account for multiple testing when performing GWAS with the same trait data and not just 1 set of SNPs called against 1 reference, but 18 additional SNP sets called against different references? If the additional SNPs were independent (and they clearly are not), then defining 5% significance with Bonferroni correction would mean dividing the original p-values not only by the number of SNPs in each set (>800k) but also by the total number of SNP sets (19). In reality, there should be a very high correlation in SNP sets (as fig S3 generally shows), but still the additional tests that are actually being performed with each new SNP set are not properly being taken into account by the Bonferroni correction. Simply, with each SNP set we expect some small amount of false positives, and by doing 19 sets of tests, we of course expect some increase in false positives, even if small.*

**RESPONSE:** As stated above, we entirely agree with this important point. Given this generally unresolved issue of multiple testing in multi-reference GWAS but also k-mer based approaches, we propose a more cautionary approach based on more stringently setting p-value threshold and discuss alternative approaches in the Discussion. In our opinion, identifying orthologous positions in divergent genomes at bp-resolution to avoid redundant loci seems a major unresolved challenge in the field as a whole.

***Part II – Major Issues: Key Experiments Required for Acceptance*** *Please use this section to detail the key new experiments or modifications of existing experiments that should be absolutely required to validate study conclusions.

Generally, there should be no more than 3 such required experiments or major modifications for a "Major Revision" recommendation. If more than 3 experiments are necessary to validate the study conclusions, then you are encouraged to recommend "Reject".*

*Reviewer #1: (No Response)*

*Reviewer #2: Although I agree with the authors that the differential detection of loci depending on the reference genome used is likely due to presence/absence variation or issues with reliably calling SNPs, I feel that the results could be bolstered by investigating this further. Are the genes in close proximity to these reference-genome specific loci truly absent in the isolates where the locus was not detected? Or if the gene is present in reference datasets where the locus was not detected, were those regions devoid of SNPs and/or aligned reads? Choosing a few of these scenarios and exploring them further may help illustrate the impact of these results a little more clearly.*

**RESPONSE:** We agree that this is a relevant issue to further illustrate. We also agree that there is unlikely to be a general solution to such a question given the complexity of structural variation present among individuals. Following the reviewer's suggestion, we now provide an illustration of the phenomenon of reference-genome specific associations for particular loci as a Supplementary Figure S3 and mentioned in the results.  *L221-234: Why limit the k-mer mapping to the IPO323 genome? One of the major novelties of this manuscript is capturing and associating unexplored genetic variation to phenotypic traits and by only mapping significant k-mers to IPO323, I feel that some important associations to novel genes may be missed. Would mapping the identified k-mers to the alternate references help place some of the unmapped k-mers? Or could k-mers be assembled into contigs and used to search the pangenome gene space? I think this would help further demonstrate the utility of this approach if all available data can be used and would also more accurately reflect the proportions of mapped/unmapped k-mers.*

**RESPONSE:** We intentionally limited the mapping approach to the IPO323 genome to reduce complexity of the presented analyses and reduce issues with redundancy (*i.e.* multiple testing issues as above). We realize that in our approach a subset of k-mers not mapped to the IPO323 genome could be better investigated by performing mapping to the 18 additional reference genomes. Our major concern with showing these analyses is that we (and any other study based on k-mers) face significant uncertainties in placing 25-bp k-mers already based on a single reference genome. Even though a significant portion remained unmapped, we also found many multi-mapping k-mers. These are most likely explained by TEs or similarly repetitive loci. Nevertheless, we have realigned the significant k-mers to a second reference genome, 1A5, which showed a similar pattern of k-mers with unique positions, multiple locations, and those that were unmappable. We have included a figure as a Supplementary Figure S5 and mentioned in the text.

*Reviewer #3: As mentioned above, the authors state using 49 traits, but although 2 supplemental tables are used to provide the raw measurements taken for the traits, the full list of 49 traits are never properly listed anywhere (even in a supplemental table). In the methods they describe how some of the trait values are calculated based on the raw measurements taken, but without the full list of 49 traits I do not know if the description of calculating any other traits is missing. Please include a full listing of the 49 traits.*

**RESPONSE:** We apologize for the lack of clarity in this regard. We now provide the full details of the 49 traits in Supplementary Table S5 and mention these more explicitly in the main text.

*Related - what is EAM (line 239 and in some supplemental tables) - is this the same as MCA? And overall virulence or overall reproduction, and reproduction specificity - these are derived phenotypes mentioned later in the manuscript but not otherwise defined within the Methods section of the paper.*

**RESPONSE:** We now clearly provide explanations for these abbreviations in the main text.

*And related to the number of traits, the authors focus their conclusions (including figures and supplemental tables) on a subset of only 20 traits, but it is never explained why - some additional explanation here is required!*

**RESPONSE:** We again regret the lack of clarity and now provide a clearer rationale for this.

*I found it odd that in table S5 there are no cases where 0 significant SNPs were recovered across all of the different reference genomes. Does this mean the authors filtered out their results to drop any cases where 0 significant SNPs were recovered in 1 or more of the reference genome analyses? Or traits were dropped because no significant SNPs were found in ANY of the reference genome analyses? Were other traits dropped, and if so, for what reason? And could any of the study's conclusions change if additional traits were included in the paper?
Please address this issue.*

**RESPONSE:** We believe that this is the same concern as stated above about what trait associations we reported. Out of the 49 traits available in total, only 20 traits showed significant GWAS associations. We now clearly document all the 49 traits as a Supplementary Table S5 for full transparency. The text now also reflects this clearly. We believe that focussing on presenting the ~40% of the phenotypic traits showing at least some significant associations out of the total available phenotypic dataset is not raising any concern about our methodology. We also believe that none of our claims rely on finding significant associations in all previously assessed traits for the GWAS population.

*The other major issue requires some discussion of multiple testing and the Bonferroni corrections used, in the context of their study design. For the most part, the value of the study is in the comparisons of the results when performing 1 individual GWAS analysis (say, just k-mer, or SNP based using only 1 particular reference) versus the results when performing a different GWAS analysis. In this case, the Bonferroni corrections are probably fine. But by suggesting that other researchers might take their approach of defining multiple SNP sets and performing GWAS across all of them in order to maximize the number of significant markers identified is statistically more problematic - here is where the Bonferroni correction as carried out is probably not quite adequate, or at least misleading, and this needs to be stated somewhere.*

**RESPONSE:** We entirely agree that a simple Bonferroni correction is not adequate and more stringent cut-offs are necessary. We discuss now this issue explicitly and suggest possible remedies.

*Perhaps there is some other way (independent of the k-mer approach) to combine SNP markers across the different SNP sets (pangenome graphs, as mentioned in line 336?) that would avoid so much redundancy in tests performed, where the total number of tests could be reduced and a better/more conservative Bonferroni correction could be applied to help reduce the chance of false positives. I am not asking the authors to alter their analyses methods in this paper to address the issue, but at a minimum they must point out the issue, so that other researchers would know not only is using multiple reference assemblies as they have done costly in effort (making the assemblies and defining each SNP set), but it would also be more statistically costly too, because it does involve performing (many) more tests.*

**RESPONSE:** We agree that a pangenome approach might be the strongest way to address proper multiple testing issues. We now include how such a pangenome could be constructed and implemented. We see though needs for improvement even in state-of-the-art pangenome graph approaches to fully resolve what redundancy exists in such datasets.

***Part III – Minor Issues: Editorial and Data Presentation Modifications*** *Please use this section for editorial suggestions as well as relatively minor modifications of existing data that would enhance clarity.*

*Reviewer #1: 1. First, I noticed that two different statistical approaches were used to account for multiple tests in GWAS. In SNP GWAS, a very stringent approach, the Bonferroni method, was used, while a permutation approach was employed for the k-mer GWAS. The Bonferroni correction method assumes that each marker is independent, which is not true and may limit the power of SNP GWAS. I am curious why the permutation approach cannot be used as well for SNP GWAS.*

**RESPONSE:** Yes, we agree that this approach is very stringent. We discuss now the use of thresholds explicitly in the discussion to also address the issue of multiple testing with multiple reference genomes.

*2. Three reasons were provided to justify the selection of 25-mer (L197-200). I think the selection of the k-mer size is related to the size of the complexity of genomes. 25-mer seems to be a reasonable selection for the small microbial genome. However, even for small genomes, the longer k-mer can facilitate accurate genomic mapping to find k-mers’ genomic location. In the justification, Reason 2 is a little hard to understand. Sequence sharing should not be a big issue among closely related genomes since the density of genomic polymorphisms among them should be low. In addition, given the high accuracy of Illumina read, sequencing errors mentioned in Reason 3 are not a big issue either as long as the k-mer length is not too long. Maybe the justification can be revised.*

**RESPONSE:** Yes, choosing k-mer length is indeed subject to trade-offs. Longer k-mers do clearly facilitate mapping back locations, however longer k-mers introduce challenges that matches are no longer perfect. We have revised the text and integrated the comments made above.

*3. Trait-associated k-mers are frequently found in TE. Because presence/absence k-mers were used as markers, here are possibilities: 1) An associated TE is present in some strains but in others; 2) A TE have many copies in all genomes (or strains) and a particular copy carrying variants was associated with traits. For the latter case, the TE copy carrying variants might insert to certain genes for phenotypic impacts. This is likely to be hard to investigate but it would be nice if more details can be provided.*

**RESPONSE:** We agree that this is an interesting challenge. We provide now more elements in the discussion but we feel unable to improve the accuracy of mapping k-mers to repetitive loci. We believe that only long-read sequencing of the GWAS panel would help clearly establish where a TE might have reinserted and influence trait expression in its new location.

*Minor comments:
4. Although genomic inflation factors were calculated and controlled, if I understand this correctly, the p-value inflation based on Q-Q plots in Figure 4 seems to be high. Please help explain.*

**RESPONSE:** We have incorporated a text in the manuscript to address this issue.

*5. Of p-values in Figure 5F and Figure 6F, it is probably not correct to make numbers after “e” be superscript, 1.6e-08 is very different from 1.6e(-08 as superscript).*

**RESPONSE:** Thank you. We have corrected the p-value notions in the Figures 5F and 6F.

*Reviewer #2: L132-133: It is not clear if GIFs were calculated for each individual reference-based GWAS (each trait/SNP dataset combination).*

**RESPONSE:** We have clarified this in the text.

*L128-149: The variation observed among the various alternative reference genome datasets is very interesting. However, instead of reporting the number of SNPs, it would be informative to report the number of distinct loci. Presumably, multiple SNPs in LD are being detected at a single locus. I suggest delimiting these results further into the number of individual loci (by examining local LD or setting an LD threshold to bin SNPs) which may more accurately reflect the number of genes detected for each trait. Although the ortholog accumulation analysis somewhat gets to this point, I still think this information should be relayed when discussing the genetic analyses.*

**RESPONSE:** We thank the reviewer for this comment and we have addressed these issues in the Discussion section.

*L483-484: Filtering for adapter sequences and quality were previously described, but how were the raw sequences filtered for contamination?*

**RESPONSE:** We have added an explanation to this comment.

*Figure 2B: The y-axes have different maximum limits. I suggest making the y-axis ranges uniform to make the visualization more comparable. Also, is the left Manhattan plot showing associations with virulence on landrace 1204? The specific line should be mentioned in the figure title and/or caption.*

**RESPONSE:** We have adjusted the scale to be proportional between different Manhattan plots. We left the maximum values untouched as these should not hinder readibility. The landrace 1204 is now mentioned specifically.

*Figure S3 was blurry and difficult to read. Also, “Pathogen” is misspelled in the upper left quadrant.*

**RESPONSE:** For fixed the spelling mistake and provide the figures as PDFs separately.

*-Some references in the Materials and Methods are cited in a different format (for example: Hartmann et al. 2018 in L426)*

**RESPONSE:** We have fixed this error in the main text.

*Reviewer #3: Please indicate in which analyses PCs were used as a factor or not! The Methods vaguely describes on what basis this decision was made, but it may be important in interpreting the results to know exactly when PCs were used or not.*

**RESPONSE:** We have added a text to clarify that all our GWAS results were obtained without principal components as covariates.

*Based on your plots, it appears the GIFs are averages across the different reference genomes? Please clarify this. Also, why not show some q-q plot comparisons, at least for a few examples of analyses using PCs and not using them - wouldn't this be more informative than only using GIF?*

**RESPONSE:** We have clarified the text and also included some q-q plot comparisons for several traits. Please see the Supplementary Figure S2.

*In table S5 and in the results - the variance of number of significant SNPs identified is reported (and emphasized). But of course we expect a higher variance when the mean is also higher. I suggest also computing the coefficient of variation (cv) in these cases, and reporting that instead. You will see that different traits end up being extreme when looking at cv instead of variance.*

**RESPONSE:** We have estimated the CV for each trait and incorporated the values in Supplementary Table S6 with a textual description in the results.

*SNP effect size was only estimated for the canonical reference - why? I suppose it appears in the manuscript just for illustrative purposes, and these effect sizes are not expected to be very different against other references, but it seems maybe not difficult to estimate all effect sizes. If you keep effect sizes only for the canonical reference, please provide a justification.*

**RESPONSE:** Yes, we reported only SNP effect sizes for the canonical reference genome. We believe that there is only value in reporting all effect sizes if there was an accurate and efficient process to map SNPs between the different reference genomes. For the reasons explained above, we do not believe that this is feasible. We mention now this issue specifically for effect sizes as well.

*Fig 3 please provide a statement that you are summing the distinct genes (or orthologous groups) across these sets of traits for the plot (not taking an average).*

**RESPONSE:** We have explicitly added this statement in the Figure 3 legend.

*Fig 2B - list cultivar 1204.*

**RESPONSE:** We have now listed the cultivar “1204” in Figure 2B.

*Fig 4 - am I correct in assuming that the blue dots (significant k-mers) always come in pairs because they represent the alternative SNP alleles (now coded as presence-absence for discrete k-mers)? If so, it would help to explicitly explain this.*

**RESPONSE:** We have clarified the meaning of blue dots in the figure legend.

*Lines 159-161: State that you are only counting a gene once even when it is significant for multiple (related) traits.*

**RESPONSE:** We have corrected this text in the manuscript.

*Fig S3 is cited before S2 – is that a problem for the journal?*

**RESPONSE:** We have corrected this.

*In figure S2A and S3, the figure label reads pathoge rather than pathogen.*

**RESPONSE:** We have corrected the figure label in the Supplementary Figure S4 and S6.

*In Fig5B and line 241 – given the gene orientation, shouldn't the text read downstream rather than upstream? (with respect to gene's transcription?)*

**RESPONSE:** We have corrected this in the main text.

*Line 251 - maybe cite Fig 6A not 6B? Because the q-q plot does not indicate from which region significant SNPs come.*

**RESPONSE:** We have corrected the figure citation in the manuscript.

*If initial SNP filtering requires genotype call rate >80%, then why is the 50% maximum missing values filter needed later?*

**RESPONSE:** We have corrected this in the manuscript.
